# Establishment of a Plasmid-Based Reverse Genetics System for the Cell Culture-Adapted Hepatitis E Virus Genotype 3c Strain 47832c

**DOI:** 10.3390/pathogens9030157

**Published:** 2020-02-25

**Authors:** Johannes Scholz, Christine Bächlein, Ashish K. Gadicherla, Alexander Falkenhagen, Simon H. Tausch, Reimar Johne

**Affiliations:** 1 Department Biological Safety, German Federal Institute for Risk Assessment, Max-Dohrn-Straße 8-10, 10589 Berlin, Germany; Johannes.Scholz@bfr.bund.de (J.S.); Ashish.Gadicherla@bfr.bund.de (A.K.G.); Alexander.Falkenhagen@bfr.bund.de (A.F.); Simon.Tausch@bfr.bund.de (S.H.T.); 2 Institute of Virology, Department of Infectious Diseases, University of Veterinary Medicine Hannover, Buenteweg 17, 30559 Hannover, Germany; Christine.Baechlein@tiho-hannover.de

**Keywords:** hepatitis E virus, genotype 3c, cell culture, reverse genetics system, site-directed mutagenesis

## Abstract

The hepatitis E virus (HEV) causes acute and chronic hepatitis in humans. Investigation of HEV replication is hampered by the lack of broadly applicable, efficient cell culture systems and tools for site-directed mutagenesis of HEV. The cell culture-adapted genotype 3c strain 47832c, which represents a typical genotype predominantly detected in Europe, has previously been used for several basic and applied research studies. Here, a plasmid-based reverse genetics system was developed for this strain, which efficiently rescued the infectious virus without the need for in vitro RNA transcription. The cotransfection of T7 RNA polymerase-expressing BSR/T7 cells with one plasmid encoding the full-length viral genome and two helper plasmids encoding vaccinia virus capping enzymes resulted in the production of infectious HEV, which could be serially passaged on A549/D3 cells. The parental and recombinant virus exhibited similar replication kinetics. A single point mutation creating an additional restriction enzyme site could be successfully introduced into the virus genome of progeny virus, indicating that the system is suitable for site-directed mutagenesis. This system is the first plasmid-based HEV reverse genetics system, as well as the first reverse genetics system for HEV genotype 3c, and should therefore be of broad use for basic and applied HEV research.

## 1. Introduction

Hepatitis E is a human disease which is highly prevalent worldwide with more than 20 million infections and over three million cases each year [1]. A wide range of symptoms, from acute self-limiting hepatitis to chronic liver inflammation in immunocompromised patients, is associated with the disease [2]. Large disease outbreaks are common in developing countries in Asia and Africa, whereas sporadic cases are predominant in industrialized countries. In Europe, the number of notified, locally acquired hepatitis E cases has steeply increased in the last decade [3].

The disease is caused by infection with the hepatitis E virus (HEV), which is classified into the genus *Orthohepevirus* within the family *Hepeviridae* [4]. HEV is fecally excreted as a nonenveloped particle, but enveloped particles have been identified in serum and cell culture supernatants [5]. The genome is comprised of one single-stranded RNA molecule of positive polarity with a length of approximately 7 kb, containing three open reading frames (ORFs). ORF1 encodes a nonstructural polyprotein, ORF2 encodes the capsid protein, and ORF3 encodes a small multifunctional phosphoprotein [4]. 

HEV can be further classified into genotypes and subtypes [6]. Genotypes 1 to 4 represent the main human-pathogenic HEV types. Genotypes 1 and 2 exclusively infect humans, whereas genotypes 3 and 4 have zoonotic potential and are widely distributed in pigs and wild boars [7]. In industrialized countries of the European and American continent, genotype 3 is the main circulating genotype, which is most likely transmitted to humans by pork products [3]. 

Research on HEV is hampered by the lack of broadly applicable and efficient cell culture systems and tools for site-directed mutagenesis. Only a few genotype 3 strains have been reported to efficiently grow in cell culture [8,9]. Among them, the genotype 3a strain Kernow-C1 and the genotype 3b strain JE03-1760F have been used in several studies [8]. For both of these strains, reverse genetics systems based on in vitro-transcribed RNA from infectious cDNA clones have been developed, which enable targeted genome manipulations [10,11]. However, for the HEV genotypes 3c, 3e, and 3f, which are predominantly detected in hepatitis E patients in Europe [12], no reverse genetics systems are currently available.

The genotype 3c strain 47832c was originally isolated from a chronically infected transplant patient in Germany [13]. It has been shown to replicate in A549 cells and even more efficiently in the subclonal cell line A549/D3 [14]. The strain has a unique insertion in its hypervariable region of the ORF1, composed of duplications from two different regions of its own ORF1, which has been speculated to be involved in efficient cell culture growth. Strain 47832c has been previously used for several research applications, such as the assessment of heat stability of HEV [15] or its susceptibility to antiviral substances [16]. However, site-directed manipulation of its genome is not currently possible.

For this reason, the aim of the study was to generate and optimize a reverse genetics system for the HEV strain 47832c. To exclude growth disadvantages induced by the cloning and virus rescue procedure, the growth kinetics of unmodified and genetically engineered viruses were compared. Finally, a point mutation was introduced to demonstrate the suitability of the system for site-directed mutagenesis.

## 2. Results

### 2.1. Generation of a Genomic Plasmid of the HEV Strain 47832c 

To generate a genomic plasmid of the virus, RNA was isolated from the cell culture supernatant of A549 cells persistently infected with HEV strain 47832c. Overlapping genome fragments were amplified by RT-PCR and subsequently cloned in a pBR322-derived vector backbone downstream of a T7 RNA polymerase promotor. Using the primer sequences specified in Supplementary Table S1, a poly A-sequence was inserted at the genome 3′-end. Restriction enzyme cleavage and Sanger sequencing confirmed the correct insertion of the HEV genome into the functional sequences of the plasmid.

**Figure 1:**
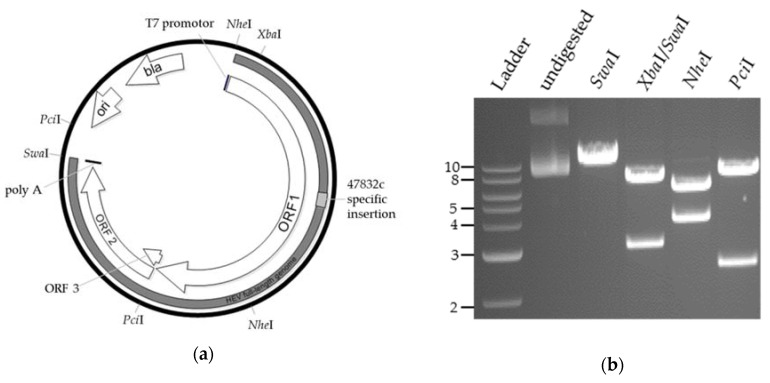
Generation of a genomic plasmid for hepatitis E virus (HEV) genotype 3c strain 47832c. (**a**) Plasmid map of p47832mc, bla = beta-lactamase gene, ori = origin of replication. (**b**) Restriction enzyme analysis of plasmid p47832mc using the indicated enzymes and subsequent agarose gel electrophoresis, Ladder: Quick-Load® 2-Log DNA-Ladder (New England Biolabs, NEB), in kbp.

Using this plasmid, several trials using direct plasmid transfection into BSR T7/5 cells or transfection of in vitro-transcribed RNA into different cell lines (A549 and A549/D3 cells) were attempted. However, no infectious virus could be generated, as indicated by the absence of ORF2-expressing cells in the first passage of culture supernatants. A comparison of the cloned HEV sequence with the sequence directly obtained from the patient’s serum and with the sequence from a low-passaged cell culture supernatant indicated differences at 24 nucleotide positions (Supplementary Results, Table S2). Of the 24 nucleotide substitutions, 12 resulted in amino acid residues that were neither present in the sequence derived from the patient’s serum nor in the sequence derived from low-passaged cell culture virus (Supplementary Results, Table S2). Therefore, genome parts with corrected nucleotides (Supplementary Results, Table S2) were synthetized and exchanged with the corresponding parts of the initial genomic plasmid, which was subsequently designated as p47832mc (i.e., plasmid 47832 molecularly cloned, Figure 1a). Restriction enzyme analysis (Figure 1b) and DNA sequencing confirmed the correct sequence of the whole plasmid.

### 2.2. Generation of Infectious HEV by Transfection of the Genomic Plasmid

**Figure 2:**
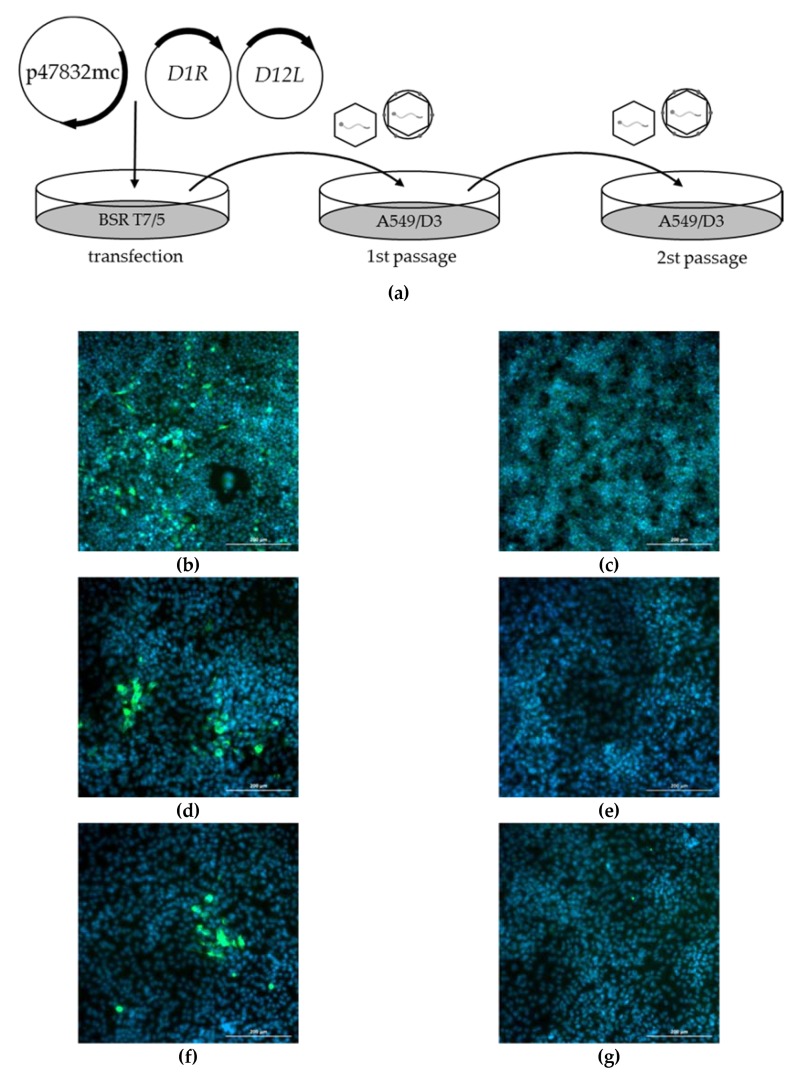
Generation of infectious HEV by transfection of the genomic plasmid p47832mc. (**a**) Schematic presentation of the procedure; circular plasmid p47832mc was cotransfected together with two plasmids expressing capping enzymes from the vaccinia virus (pCAG D1R and pCAG D12L). After 7 d of incubation, the supernatant was used to infect A549/D3 cells. After 14 d of incubation, this supernatant was used for a second passage on A549/D3 cells, again incubated for 14 d. (**b–g**) Immunofluorescence analysis of cells using an HEV capsid protein-specific antiserum; green signal HEV-specific, blue: 4′,6-diamidino-2-phenylindole DAPI stain. Scale bar 200 µm. (**b**) BSR T7/5 cells at 7 d after transfection; (**c**) nontransfected BSR T7/5 cells; (**d**) A549/D3 cells at 14 d after infection with supernatant of transfected cells; (**e**) A549/D3 cells at 14 d after inoculation with supernatant of nontransfected cells; (**f**) A549/D3 cells at 14 d after infection with supernatant from first passage; (**g**) A549/D3 cells at 14 d after infection with supernatant from noninfected first passage.

After redesigning the genomic plasmid, we decided to focus on the generation of recombinant HEV by direct plasmid transfection to avoid the need for in vitro RNA transcription followed by RNA transfection. This required the presence of T7 RNA polymerase in transfected cells to ensure the exact start of the transcription of the viral genome. For optimization of the plasmid transfection and virus passaging system, several parameters (different cell lines, transfection reagents, culture media, incubation times, temperatures, and plasmid-mediated T7 RNA polymerase expression) were tested. The final protocol of the optimized procedure is described in the Materials and Methods Section and is schematically shown in Figure 2a. Briefly, plasmid p47832mc was cotransfected together with two helper plasmids expressing subunits of the vaccinia virus capping enzyme into BSR T7/5 cells. The T7 RNA polymerase, which is constitutively expressed in these cells, transcribes the plasmid p47832mc, generating a complete HEV genomic RNA which can be capped by the helper plasmid products and translated by the cellular machinery. 

Analysis of the transfected BSR T7/5 cells at 7 d after transfection by immunofluorescence using an HEV capsid protein-specific antiserum revealed the presence of many single fluorescent cells (Figure 2b). Thereafter, the supernatant of the cells was used for the infection of A549/D3 cells, which are known to be susceptible for strain 47832c. Several fluorescent foci were identified by immunofluorescence at 14 d after infection (Figure 2d). Fluorescent foci were also observed after a second passage in D549/D3 cells (Figure 2f), proving the production of infectious HEV following transfection of BSR T7/5 cells. The experiment was repeated three times with similar results.

### 2.3. Characterization of the Generated Virus

**Figure 3:**
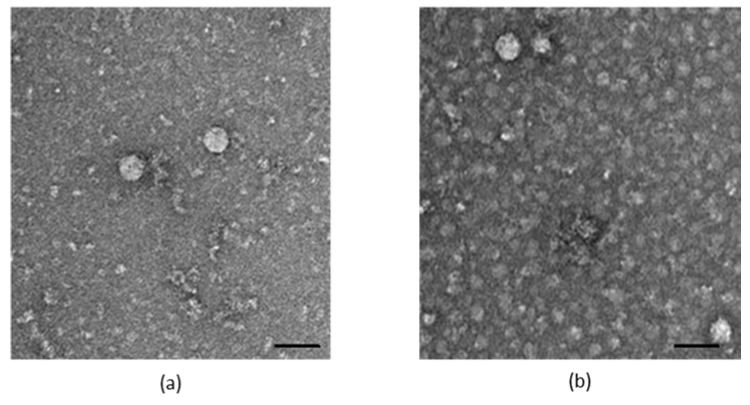
Morphological analysis of the generated virus 47832mc using transmission electron microscopy (TEM). Supernatant of the fifth passage was used for TEM imaging; (**a)** wild-type HEV; (**b**) 47832mc. Scale bar 50 nm.

The particle morphology and the genome of the generated virus (designated as 47832mc) were further analyzed. Using electron microscopy, virus particles mainly resembling the morphology of enveloped HEV particles were identified in the supernatants (Figure 3).

**Figure 4:**
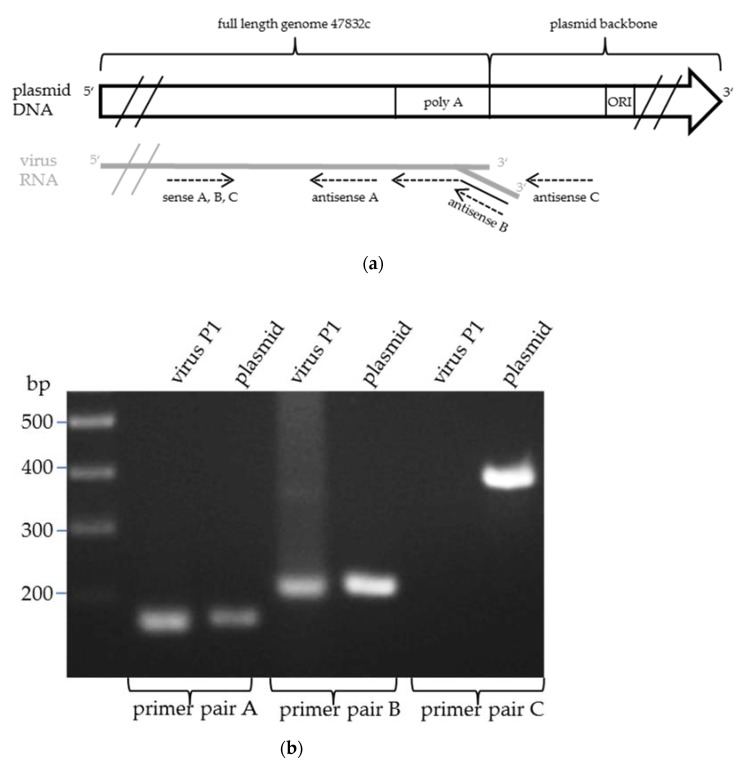
Analysis of the 3′-end of the genome of virus 47832mc using (RT)-PCR. (**a**) Schematic presentation of the primer binding sites on the plasmid and the HEV genome; forward primers A, B, C target the ORF2 region. Reverse primer A targets a region in the 3’-UTR. Reverse primer set B targets the poly-A tail. Reverse primer C targets the plasmid backbone downstream of the poly-A tail. (**b**) Agarose gel analysis after electrophoresis of the PCR products. RNA from the culture supernatant of the 1st passage of virus 47832mc (virus P1) or DNA of plasmid p47832mc (plasmid) was used. PCR product sizes: 184 bp (primer pair A), 230 bp (primer pair B), 387 bp (primer pair C).

Since circular plasmid DNA served as the template for the transcription of the viral genome in BSR T7/5 cells, the viral RNA may contain sequences at the 3′-end that were derived from the plasmid backbone. A combination of specific primer pairs was used to determine whether the 3′-end of the viral RNA contained plasmid-derived sequences (Figure 4a). As expected, using a sense primer with a binding site approximately 200 bp upstream of the poly-A-tail and antisense primers binding immediately upstream or directly at the poly-A-tail, (RT-)PCR products could be generated for both the plasmid and the resulting virus (Figure 4b, lanes 1–4). In contrast, using the same sense primer with an antisense primer binding at the plasmid sequence directly downstream of the poly-A-tail, a (RT-)PCR product was only produced with the plasmid, but not with the virus RNA as template (Figure 4b, lanes 5–6), indicating that no plasmid-derived sequences are added to the 3′-end of the generated virus genome.

**Figure 5:**
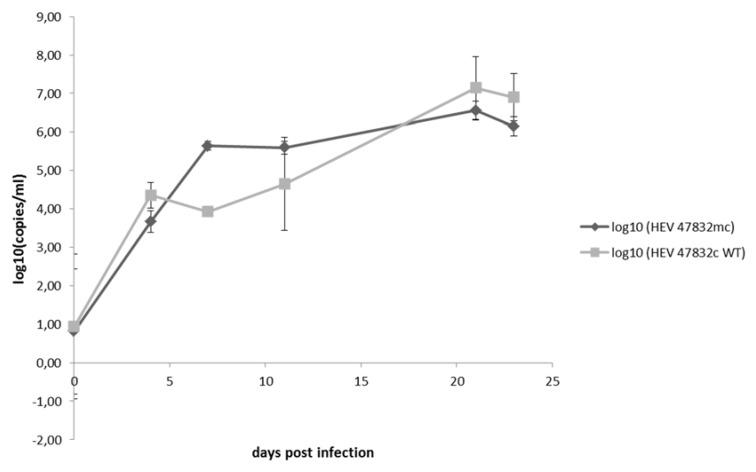
Comparison of growth kinetics of strains 47832mc generated by reverse genetics (HEV 47832mc) and the wild-type strain 47832c derived from persistently infected cells (HEV 47832c WT). A549/D3 cells were infected and supernatants were tested at the indicated time points after infection using real-time qRT-PCR. Data are means +/- standard deviation from two independent experiments.

A comparison of growth kinetics of the generated virus 47832mc with the wild-type virus 47832c derived from the persistently infected cell line was done by infection of A549/D3 cells with the same infectious doses and subsequent testing of the supernatants for HEV RNA by quantitative RT-qPCR. As shown in Figure 5, similar increases in viral RNA genome copies over a period of 24 d are evident. 

### 2.4. Introduction of a Point Mutation into the Virus Genome

**Figure 6:**
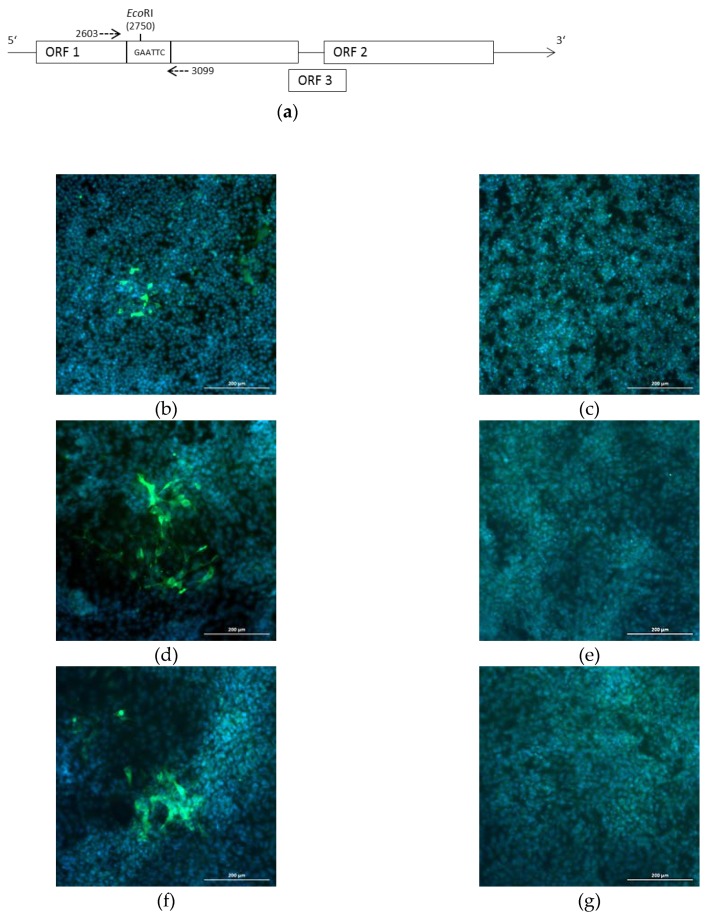
Generation of plasmid p47832*_Eco_*_RI_ harboring a single nucleotide exchange, which generated an additional *Eco*RI site in the virus genome. (**a**) Linear scheme of plasmid p47832*_Eco_*_RI_, dotted arrows indicate primer binding sites for the analytical RT-PCR; (**b–g**) immunofluorescence analysis of cells using an HEV capsid protein-specific antiserum; green signal HEV-specific, blue: DAPI stain. Scale bar 200 µm. (**b**) BSR T7/5 cells at 7 d after transfection with p47832*_Eco_*_RI_; (**c**) nontransfected BSR T7/5 cells; (**d**) A549/D3 cells at 14 d after infection with supernatant of transfected cells; (**e**) A549/D3 cells at 14 d after inoculation with supernatant of nontransfected cells; (**f**) A549/D3 cells at 14 d after infection with supernatant from first passage; (**g**) A549/D3 cells at 14 d after infection with supernatant from noninfected first passage.

**Figure 7:**
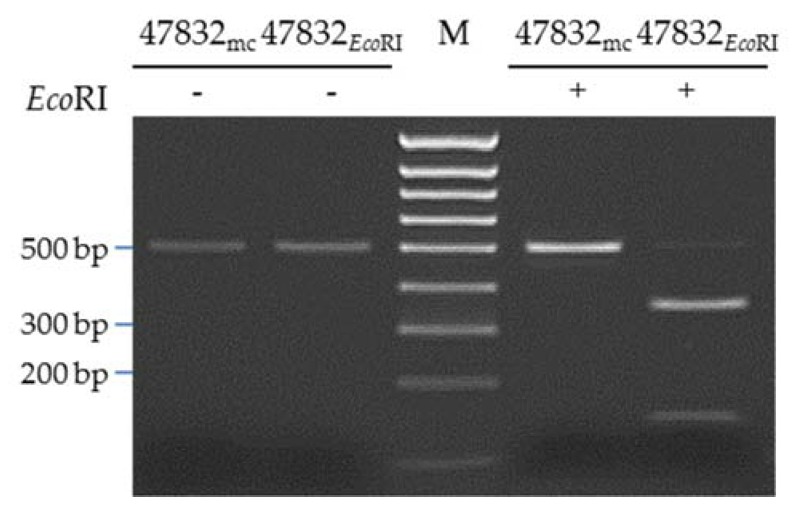
Analysis of the generated virus genomes for the presence of the introduced *Eco*RI site. Supernatants of the first virus passage in A549/D3 cells after transfection of BSR T7/5 cells with plasmid p47832mc or p47832*_Eco_*_RI_ were analyzed by RT-PCR, generating a 497-bp product. RT-PCR products without (left) or with (right) *Eco*RI treatment are shown.

To show the suitability of the system for site-directed mutagenesis, a silent point mutation resulting in a newly generated *Eco*RI restriction site was introduced into plasmid p47832mc, resulting in plasmid p47832_EcoRI_. The position of the nucleotide substitution within the insertion in ORF1 of the genome is shown in Figure 6a. After transfection of p47832_EcoRI_ into BSR T7/5 cells and subsequent two passages of the supernatant in A549/D3 cells, fluorescent staining similar to p47832mc was evident (Figure 6b–g). Analysis of the genetically modified virus from passage 1 by RT-PCR amplification and restriction enzyme digestion with *Eco*RI confirmed the presence of the point mutation in the virus genome (Figure 7). 

## 3. Discussion

Reverse genetics systems represent powerful tools for basic and applied studies in virology. For HEV, such applications have been developed only for a few cell culture-adapted strains so far. Here, we presented the construction of a reverse genetics system for the HEV genotype 3c, which represents a highly prevalent genotype in Europe [12]. Our system is based on the HEV strain 47832c, which has been already used for several applied studies, thus confirming its suitability for research on HEV [9,15,16]. The strain harbors a specific genome insertion resulting from duplications of parts of its genome, which might confer efficient cell culture growth [13]. Another HEV strain very frequently used for studies involving cell culture and site-directed mutagenesis is the genotype 3a strain Kernow-C1 [8]. This strain also harbors an insertion of similar location and size as compared to strain 47832c. However, the insertion in strain Kernow-C1 is derived from a human ribosomal protein gene [10,17], which might involve specific human protein interactions not present in other HEV strains. The significance and functions of the insertions in both strains should be analyzed and compared to each other in future studies to elucidate the mechanisms conferring efficient HEV replication in cell culture. 

The first version of the plasmid containing the full-length genomic sequence of strain 47832c did not lead to successful generation of the infectious virus. Only a whole genome sequence comparison with the original viral sequences from the infected patient and from a lower passaged cell culture isolate enabled the identification of unique single nucleotide mutations in the plasmid. After these were corrected, the infectious virus was readily generated. The occurrence of nucleotide mutations, which prevented efficient generation of infectious virus, has also been described for the first versions of cDNA clones of HEV genotype 1 strain SAR-55, the genotype 3 strain Meng and for an avian HEV strain [18,19,20]. For generation of the fast replicating cDNA clone of the Kernow C1 strain p6, a stepwise cloning method exchanging genome segments from a slowly replicating cDNA version with newly amplified PCR products followed by the functional testing of each chimeric clone had to be applied [10]. Mutations naturally occurring during virus replication in cell culture or sequence errors introduced by RT-PCR may be responsible for the observed mutations, which resulted in decreased replication efficiency.

Using the improved plasmid and an optimized protocol, infectious HEV could be readily rescued, as demonstrated by the immunofluorescence analysis of cells infected with the first and second passage virus. It should be noted that the protocol applied in this study was not dependent on the use of in vitro-transcribed RNA, in vitro RNA capping, or RNA transfection, as described for the other reverse genetics systems published so far for HEV [10,11,18,19,20]. Handling of RNA is generally difficult because of the possible presence of RNases in the samples. In addition, in vitro transcription, capping, and transfection of RNA may be less efficient as compared to a single transfection with plasmid DNA. The general principle of the system used by us, in which the genomic plasmid is directly transfected together with two capping helper plasmids into T7 polymerase-expressing cells, was first described for rotaviruses [21]. Thus, the application of this system onto HEV resulted in a robust and easy-to-handle reverse genetics system. Although no restriction enzyme cutting or ribozyme cleavage was used, we proved that the 3’ viral genomic end was correctly engineered using this method. Other mechanisms, e.g., during generation of the antisense RNA by the HEV polymerase, are supposed to be responsible for the correct processing of the HEV genome end. Efforts should be made in the future to further optimize the system by testing other promoters and cell lines that do not require co-expression of the T7 RNA polymerase or the vaccinia virus capping enzymes.

The system was further validated. By comparison of the growth curves of the generated virus and the wild-type strain derived from persistently infected cells, no obvious differences were found. Furthermore, a single nucleotide exchange leading to a unique restriction site could be successfully introduced into the recombinant virus genome. This demonstrates the suitability of the system for site-directed mutagenesis, which might be used in future studies for investigating the effect of single nucleotide mutations on viral fitness, e.g., mutation G1634R, which has been described to increase the replication efficiency in other HEV strains [22,23].

In conclusion, a robust reverse genetics system was developed here for the cell culture-adapted HEV genotype 3c strain 47832c. The suitability of the system for site-directed mutagenesis was demonstrated. Future applications of the system may include research on basic questions concerning HEV replication, e.g., the functional analyses of the genome insertion in strain 47832c, and may pave the way for translational studies, such as targeting effects of single nucleotide exchanges on the drug resistance of HEV.

## 4. Materials and Methods 

### 4.1. Cells and Viruses

The cell line A549/D3 is a subclone of the human lung adenocarcinoma cell line A549, which shows a higher susceptibility to infection with the HEV strain 47832c [14]. The A549 cell line persistently infected with the HEV strain 47832c has been described by Johne et al. [13]. The T7-polymerase expressing hamster kidney cell line BSR T7/5 [24] was kindly provided by Karsten Tischer (Free University of Berlin, Berlin, Germany) and maintained in medium containing 1 mg/mL G418 (Biochrom, Berlin, Germany). The HEV strain 47832c is a genotype 3c strain originally isolated in A549 cells from a chronical infected patient from Germany [13].

### 4.2. Generation of the Genomic Plasmid p47832_mc_

RNA was manually extracted from the supernatant of A549 cells persistently infected with HEV strain 47832c [13] using the Viral RNA Mini Kit (Qiagen, Hilden, Germany). Primers listed in Table S1 were used to generate HEV-specific amplicons with 5’- and 3′-end overhangs, which overlapped with the plasmid up- and downstream at the site of insertion. Reverse transcription was carried out either with Moloney Murine Leukemia Virus (M-MLV)- (Invitrogen, Carlsbad, CA, USA) or with SuperScript II reverse transcriptase (Thermo Fisher Scientific, Waltham, MA, USA) and the following PCR using the Phusion High-Fidelity Polymerase (NEB). For cloning of the complete HEV genome, a restriction-free strategy was applied. Briefly, the amplicon generated during the first PCR was used as a primer in a second amplification reaction with the plasmid vector serving as a template. The resulting PCR product was digested with 1 µL of *Dpn*I (NEB) and 5 µL of the reaction mix was used to transform chemically competent *E. coli* HB101 cells. Positive clones were selected with Ampicillin (100 µg/mL) and were grown in LB-Medium. 

To generate an HEV genomic plasmid with corrected point mutations, parts of the HEV genome were newly synthetized, combined with parts of the already available genomic clone, and recloned into a new vector backbone. Briefly, the plasmids p47832fc1 and p47832fc2, containing fragments of the HEV47832c genome with the nucleotide substitutions listed in Supplementary Results, Table S2, were synthesized by Integraded DNA Technologies, Inc. (IDT, Newark, NJ, USA). The fragment in p47832fc1 corresponded to the *Hin*dIII/*Mre*I genome fragment of HEV strain 47832c, while the fragment in p47832fc2 corresponded to the HEV strain 47832c genome region ranging from the *Pci*I restriction site to the poly-A- tail at the 3′-end followed by *Swa*I and *Pci*I restriction sites. The plasmid p47832mc was constructed in four cloning steps. First, the *Nhe*I/*Hin*dIII fragment of the original HEV genomic plasmid was replaced by a *Nhe*I/*Hin*dIII fragment of pT7-VP1SA11 (Addgene plasmid #89162; a gift from Takeshi Kobayashi) (Kanai, Komoto et al. 2017), resulting in plasmid p47832xc1. Thereafter, a *Mre*I/*Pci*I fragment of the original HEV genomic plasmid was inserted into the corresponding restriction sites of p47832fc1, resulting in plasmid p47832xc2. In the third cloning step, a *Hin*dIII/*Pci*I fragment of p47832xc2 was replaced by the *Hin*dIII/*Pci*I fragment of p47832xc1, resulting in plasmid p47832xc3. Thereafter, the *Pci*I/*Pci*I fragment of p47832fc2 was inserted into the *Pci*I site of p47832xc3, resulting in the final plasmid p47832mc.

For sequencing of the whole plasmid p47832mc, the plasmid was purified using a plasmid midi kit (Qiagen, Hilden, Germany) and concentration was measured by a NanoDrop 1000 device (Thermo Fisher Scientific, Waltham, MA, USA). Thereafter, a library was prepared for Illumina sequencing using the Nextera DNA Flex kit (Illumina, San Diego, CA, USA). A total of 705,204 paired-end reads were sequenced on a NextSeq instrument (Illumina, San Diego, CA, USA). Read trimming using fastp v. 0.19.5 [25] with default parameters and a minimum read length of 15 bp resulted in 677,764 read pairs. An assembly of these reads using the assembly pipeline shovill v. 1.0.4 (unpublished, https://github.com/tseemann/shovill) based on the assembly algorithm spades v. 3.13.1 [26] resulted in a plasmid contig with 1826 × coverage. The sequence of plasmid p47832mc was submitted to the GenBank database with the accession number MN756606.

### 4.3. Generation of the Mutated Plasmid p47832_EcoRI_

For the introduction of the *Eco*RI restriction site, a gene fragment corresponding to the *Bsr*GI/*Hpa*I fragment of p47832fc1 and carrying the nucleotide exchange T1234C was synthesized by IDT (Newark, NJ, USA). The plasmid p47832*_Eco_*_RI_ was constructed in two cloning steps. First, the *BsrG*I/*Hpa*I fragment of p47832fc1 was replaced by the synthesized gene fragment. Thereafter, a *Hin*dIII/*Mre*I fragment of the resulting plasmid was replaced with the corresponding fragment from plasmid p47832mc, resulting in plasmid p47832*_Eco_*_RI_. The sequence of plasmid p47832*_Eco_*_RI_ was submitted to the GenBank database with the accession number MN756607.

### 4.4. Generation of Infectious Virus from Plasmids

At 24 h before transfection, 8 × 10^5^ BSR T7/5 cells/well were seeded in a six-well plate and incubated in fresh Dulbecco’s modified Eagle medium (DMEM) supplemented with 10% fetal calf serum (FCS), 1% nonessential amino acids (NEAA), 1% L-glutamine and 1% gentamicin (all cell culture reagents provided by Pan-Biotech GmbH, Aidenbach, Germany). All plasmids were purified using a plasmid midi kit (Qiagen, Hilden, Germany), and concentration was measured by a NanoDrop 1000 device (Thermo Fisher Scientific, Waltham, MA, USA). A total of 8 µg of plasmid DNA (p47832mc or p47832*_Eco_*_RI_) was mixed with 1 µg of pCAG D1R and 1 µg of pCAG D12L (Addgene plasmids #89160 and #89161) [21], as well as 250 µL OptiMEM (Thermo Fisher Scientific, Waltham, MA, USA) and 30 µL TransIT®-LT1 transfection reagent (MIRUS Bio, Madison, WI, USA). After incubation for 25 min, the mixture was added directly to the supernatant of BSR T7/5 cells in a six-well plate. The cells were incubated with DMEM medium supplied with 10% FCS, 1% NEAA, 1% L-glutamine and 1% gentamicin. At 24 h after transfection, total medium was changed to DMEM medium supplemented with 2% FCS, 1% NEAA, 1% L-glutamine, and 1% gentamicin followed by incubation at 34.5 °C and 5% CO_2_.

Supernatants of transfected BSR T7/5 cells taken at 7 d after transfection were used to infect fresh A549/D3 cells. Confluent (14 d after seeding) monolayers of A549/D3 cells grown in six-well plates were washed twice with deficient phosphate-buffered saline (DPBS). Thereafter, the cells were inoculated with 2 mL of the supernatant from transfected BSRT7/5 cells and incubated for 1 h at room temperature. The virus-containing supernatants were removed and the fresh DMEM medium was supplemented with 10% FCS, 1% NEAA, 1% L-glutamine, and 1% gentamicin. The cells were incubated at 37 °C and 5% CO_2_ for 14 d with one complete medium exchange after 7 d. A second passage was similarly performed using the supernatant of the inoculated A549/D3 cells for infection of fresh A549/D3 cells.

### 4.5. Immunofluorescence Analysis

Immunofluorescence analysis of the infected cells was performed as described before [15]. Briefly, cells were fixed for 30 min at 4 °C using a 1:1 acetone/methanol solution. After washing of cells with DPBS and subsequent blocking with DPBS containing 1% FCS for 1 h at 37 °C, cells were stained with an HEV capsid protein-specific rabbit hyperimmune serum (FLI, kindly provided from R. Ulrich, 1:500 in DPBS/ 1% FCS) for 1 h at 37 °C. After three washing steps with DPBS, cell were incubated with fluorescein isothiocyanate (FITC)-conjugated anti-rabbit immunoglobulin G antibody (SIGMA, 1:1000 in DPBS/ 1% FCS) for 1 h at 37 °C. Cells were washed twice with PBS and once with distillated water, and finally mounted with aqueous Roti®-Mount Fluor Care DAPI (ROTH). Fluorescence analysis was performed using the Zeiss Axio Observer Z1 microscope (ZEISS).

### 4.6. Electron Microscopy

A total of 10 µL of cell culture supernatant was adsorbed on to 400 mesh carbon-formvar coated copper grids (Plano GmbH, Wetzlar, Germany) for 5 min followed by inactivation with glutaraldehyde for a further 1 min. Excess liquid was removed by passive capillary action using a tissue paper. The grids were then contrasted with 2% uranyl acetate for 1 min and excess liquid was removed as before. The grids were allowed to dry and examined in a Jeol 1400 Plus TEM (Jeol, Tokyo, Japan), operated at 120 kV. Six different areas of the grid were examined to check the homogeneity of sample. Imaging was performed with Olympus Veleta G2 camera (EMSIS, Muenster, Germany). Particle diameter was measured using ITEM software provided by Olympus.

### 4.7. Growth Kinetics of Viruses 

For the determination of the infectious titer of the generated virus preparations, an endpoint dilution assay was performed as described earlier [15]. Briefly, A549/D3 cells were seeded in 96-well plates at 14 d prior infection and grown in Eagle’s minimum essential medium (EMEM) supplied with 10% FCS, 1% NEAA, 1% L-glutamine, and 1% gentamicin, with one complete medium exchange after 7 d. The confluent cells were washed twice with DPBS and the HEV-containing supernatants (each sample in duplicates) were added in a 10-fold dilution series. After 1 h of incubation at room temperature, the HEV supernatants were removed and fresh medium (as above) was added. The cells were incubated for 14 d at 34.5 °C and 5% CO_2_, with one complete medium exchange after 7 d. The cells were analyzed by immunofluorescence as described above and the focus-forming units (ffu)/ mL were determined.

The HEV-containing preparations were diluted to equal viral titers of 2.75 × 10^2^ ffu/mL using fresh DMEM medium supplemented with 10% FCS, 1% NEAA, 1% L-glutamine and 1% gentamicin. The infection of A549/D3 cells was performed in six-well plates as described above. The infected cells were incubated for 24 d in fresh DMEM medium supplied with 10% FCS, 1% NEAA, 1% L-glutamine, and 1% gentamicin at 37 °C and 5% CO_2_, with complete medium exchanges each 3–4 d. At the indicated time points, 500-µL aliquots of the supernatants were subjected to nucleic acid isolation using the NucliSens® EasyMag® system (Biomérieux, Marcy-l’Étoile, France). The nucleic acids were eluted into 60 µL. Thereafter, 20 µL of the eluates were digested with DNase I (Roche) at 37 °C for 4 h. The DNAse was inactivated by incubation at 75 °C for 5 min. The samples were thereafter centrifuged at 14,000 × *g* for 10 min at 4 °C and the supernatant was used for qRT-PCR analysis. Quantification of HEV RNA copy numbers was performed using a real-time RT-PCR [27] together with an in-house HEV RNA standard as described elsewhere [28]. 

### 4.8. PCR Analysis of the 3′-end of the Virus Genome 

RNA was isolated from supernatants of infected cells using the QIAamp viral RNA mini kit (Qiagen, Hilden, Germany) and thereafter analyzed by RT-PCR. The primers used for determination of the 3′-end of the generated virus genome are listed in Supplementary Results, Table S1. Briefly, three PCR primer pairs were designed, which used the same forward primer with a binding site within the 3’-region of the HEV virus genome but different reverse primers. The reverse primer A bound downstream the forward primer, but also bound within the 3’-region of the HEV virus genome. Reverse primer B bound to the polyA-tail of the HEV genome, whereas the reverse primer C bound in the plasmid region of p47832c downstream to the cloned viral genome. The QIAGEN LongRange 2Step RT-PCR Kit (Qiagen, Hilden, Germany) was used for the RT-PCR amplifications. The PCR products were analyzed by electrophoresis on ethidium bromide-stained agarose gels.

### 4.9. PCR and Restriction Fragment Analysis of Mutant 47832_EcoRI_

The virus genome region containing the *Eco*RI site in the mutant 47832*_Eco_*_RI_ was amplified by RT-PCR using primers as listed in Supplementary Results, Table S1, followed by restriction enzyme analysis. Briefly, RNA was isolated from supernatants of infected cells using the QIAamp viral RNA mini kit (Qiagen, Hilden, Germany), and the QIAGEN OneStep RT-PCR Kit (Qiagen, Hilden, Germany) was used for RT-PCR. The PCR products were purified using the QIAquick Gel Extraction Kit (Qiagen, Hilden, Germany). Thereafter, the FastDigest *Eco*RI (Thermo Fisher Scientific, Waltham, MA, USA) was added together with the FastDigest buffer (Thermo Fisher Scientific, Waltham, MA, USA) and incubated at 37 °C for 15 min. The products were analyzed by electrophoresis on ethidium bromide-stained agarose gels. 

## Supplementary Materials

The following are available online at https://www.mdpi.com/2076-0817/9/3/157/s1, Table S1: Primers used in the study, Table S2: Nucleotide exchanges identified between the original genomic plasmid, the HEV in patient serum, the HEV after cell culture isolation and the final genomic plasmid p47832mc.
