# Supplementary material for: Establishment of a Plasmid-Based Reverse Genetics System for the Cell Culture-Adapted Hepatitis E Virus Genotype 3c Strain 47832c
Source: Pathogens. 2020 Feb 25;9(3):157. doi: 10.3390/pathogens9030157 (PMC7157446; doi:10.3390/pathogens9030157)
Supplement: Supplementary file 1 [file pathogens-09-00157-s001.pdf]

Supplementary Materials:

Supplementary Table S1. Primers used in the study.

Amplicon	Forward primer (5′-3′)*	Reverse primer (5′-3′)[1]
Genome cloning (fragment 1)	GATATACATATGGCTAGCTAATACGA<br>CTCACTATAGCAGACCACGTATGTGGT	TGGCCCTTTCGTCTTCAAGAATTAATT<br>CCGCCTTCTTAAGAAATGTCCTACAAC
Genome cloning (fragment 2)	GCCGTTGTAGGACATTTCTTAAGA<br>AGGCTGTGGGTAAGTTCTGCTGTT	TGGCCCTTTCGTCTTCAAGAATTA<br>ATTCCAGCAGCATCGATCTCCAGT
Genome cloning (fragment 3)	CCAACCTAGCACTGGAGATCGATGC<br>TGCTACGGAGGTCGGCCGGGCTTG	TGGCCCTTTCGTCTTCAAGAATTAA<br>TTCCTACACACTCGAGACCAAGGG
Genome cloning (fragment 4)	CCCTTGGTCTCGAGTGTGTAGTCAT<br>GGAGGAATGTGGCATGCCCCAGTG	CGTCTTCAAGAATTAATTCCATTCG<br>TACCGGTGAAGTGCAGGTCCTTCA
Genome cloning (fragment 5)	ATGAAGGACCTGGACTTCACCGGTAC<br>GAATGGGGTCGGTGAGGTGGGTCGT	CTGGCCCTTTCGTCTTCAAGAATTA<br>ATTCCGCGCACGCGTTTTTTTTTT
Determination of 3′end (pair A)	ACTTTTGATGATTTCTGCCCG	GGAGCGCGAAACGCAGA
Determination of 3′end (pair B)	ACTTTTGATGATTTCTGCCCG	CCGAATTCCCGGGATCCTTTTTTTTTTT<br>TTTTTTV (reverse transcription)<br>CCGAATTCCCGGGATCC (PCR)
Determination of 3′end (pair C)	ACTTTTGATGATTTCTGCCCG	ATGTGAGTTAGCTCACTCATTAGGC
Analysis of *Eco*RI site	TGTACACCCGGACATGGTCA	CCCTACATACACCTTTGCCCC

[1]Nucleotides binding to the parental plasmid in the second PCR reaction are underlined.

Supplementary Table 2: Nucleotide exchanges identified between the initial genomic clone, HEV in patient serum, HEV after cell culture isolation (low passage) and the final genomic plasmid p47832mc.

Nucleotide position	Patient serum	Low-passaged cell culture virus	Genomic clone	Amino acids	Corrected genomic plasmid[1]
37	C	C	T	Silent	T
589	T	C	T	Silent	T
1081	T	T	C	Silent	C
2072	T	T	C	S683P (ORF1)	**T**
2144	A	A	G	T707A (ORF1)	**A**
2409	T	T	C	V795A (ORF1)	**T**
2833	C	C	A	Silent (ORF1)	**C**
2850	C	C	T	A942V (ORF1)	**C**
2926	C	C	T	Silent (ORF1)	**C**
2966	G	G	A	G981E (ORF1)	**G**
3234	G	G	A	R1070Q (ORF1)	**G**
3372	A	A	G	Q1116R (ORF1)	**A**
4261	C	C	T	Silent (ORF1)	T
4918	C	T	C	Silent (ORF1)	C
5574	G	A	G	R73G (ORF2)<br>Silent (ORF3)	**A**
5637	C	C	T	P94S (ORF2)<br>Silent (ORF3)	**C**
5855	T	T	C	Silent (ORF2)	C
6176	T	C	T	Silent (ORF2)	T
6263	C	C	T	Silent (ORF2)	**C**
6337	C	C	T	A327V (ORF2)	**C**
6417	C	C	G	H354D (ORF2)	**C**
6960	A	A	G	T535A (ORF2)	**A**
**7065**	C	T	C	Silent (ORF2)	C
**7315**	A	A	G	K653R (ORF2)	**A**

[1]Exchanged nucleotides in p47832mc are indicated in bold face.
